# 1q25.3–q32.1 deletion causing multisystem developmental delay: a case report and literature review

**DOI:** 10.3389/fped.2026.1774631

**Published:** 2026-06-10

**Authors:** Lifang Liu, Rong Yu, Weizhong Zhang, Shiwen Huang, Taiwei Huang

**Affiliations:** Department of Neonatology, Huizhou First Maternal and Child Health Care Hospital, Guangdong, China

**Keywords:** 1q25.3–q32.1 deletion, chromosomal microarray analysis, developmental delay, intrauterine growth restriction, language delay

## Abstract

**Objective:**

This study provides a detailed report of a pediatric patient with a 1q25.3–q32.1 deletion, reports the results of a retrospective analysis of relevant literature, and examines relevant genotype–phenotype correlations. Its objectives are to enhance clinicians’ awareness of this rare disorder and to provide clinical evidence for genotype–phenotype correlation analysis.

**Methods:**

We collected clinical data from one case of 1q25.3–q32.1 deletion, including perinatal history, clinical manifestations, auxiliary examinations, diagnosis and treatment, and follow-up information, and conducted analysis. Simultaneously, we searched literature databases, including PubMed, Embase, the China National Knowledge Infrastructure, and Wanfang, to collect domestic and international papers on 1q25–q32 deletions published up to January 2025. Clinical characteristics, genetic information, and prognostic data from these case reports were extracted and compiled to analyze genotype–phenotype associations.

**Results:**

A male newborn was born at 37 weeks’ gestation with intrauterine growth restrictions. Neonatal presentation included feeding difficulties and decreased spontaneous activity. Karyotype analysis showed 46,XY,del(1)(q23.3q25.3). Chromosomal microarray analysis revealed a pathogenic 27.0-Mb deletion in the 1q25.3–q32.1 region. Follow-up revealed persistent postnatal growth and motor developmental delays, along with language development delays, in the patient. A literature review yielded 31 cases (including this case) of 1q25–q32-deletion syndrome. Primary common clinical features included intrauterine growth restriction, postnatal growth retardation, microcephaly, facial dysmorphism, neurological abnormalities, delayed motor and language development. The deleted segment ranged from 1.5 to 28.0 Mb in size. Key genes within the deleted region (e.g., *CENPL*, *LHX4*, *DNM3*, *PBX1*) were closely associated with the clinical phenotype.

**Conclusion:**

Deletion of 1q25.3–q32.1 is a rare chromosomal rearrangement associated with corresponding phenotypic features. When children present with intellectual disability, intrauterine growth restriction, postnatal growth retardation, language developmental delays, motor developmental delays, microcephaly, facial dysmorphisms, small hands and feet, external genital anomalies, brachydactyly, and clinodactyly of the fifth finger, clinicians should highly suspect 1q intermediate chromosome deletion. It is recommended to perform chromosomal microarray analysis (CMA) as early as possible to establish a clear diagnosis, and implement individualized intervention and long-term follow-up so as to improve the prognosis. This report illustrates how molecular delineation associated with fine clinical characterization can improve the genotype–phenotype correlations of classical cytogenetic abnormalities.

## 1 Introduction

Chromosome 1 is the largest chromosome in the human genome, spanning approximately 219 Mb in length. It contains 2,057 protein-coding genes, accounting for 8% of the total human genome (1).

Chromosomal abnormalities involving 1q (the long arm of chromosome 1) are relatively rare, and, due to the varying sizes and locations of the deleted segments, the associated clinical phenotypes exhibit high heterogeneity (2). In 1982, Taysi et al. (3) classified 1q deletions into three categories based on their location: proximal deletions (1q21-22–1q25), intermediate deletions (1q24-25–1q32), and distal deletions (1q42-43–1qter). Among these, intermediate deletions (1q25–q32) are extremely rare, with only about 40 cases reported worldwide to date (4). Patients with 1q25–q32 deletions often present with multisystem involvement, such as intrauterine growth restriction, postnatal growth retardation, microcephaly, facial dysmorphism, neurodevelopmental delays, and endocrine abnormalities (4).The loss of key genes such as *LHX4*, *CENPL*, and *PRRX1* in the deleted region is closely associated with the development of clinical phenotypes (5). This study provides a foundation for elucidating the genotype–phenotype correlation of 1q25–q32 deletion by conducting a retrospective analysis of the syndrome's clinical manifestations, diagnostic approaches, and genetic findings, which is combined with a comprehensive literature review to summarize key clinical and genetic features.

## 2 Case report

### 2.1 Case description

**Figure 1:**
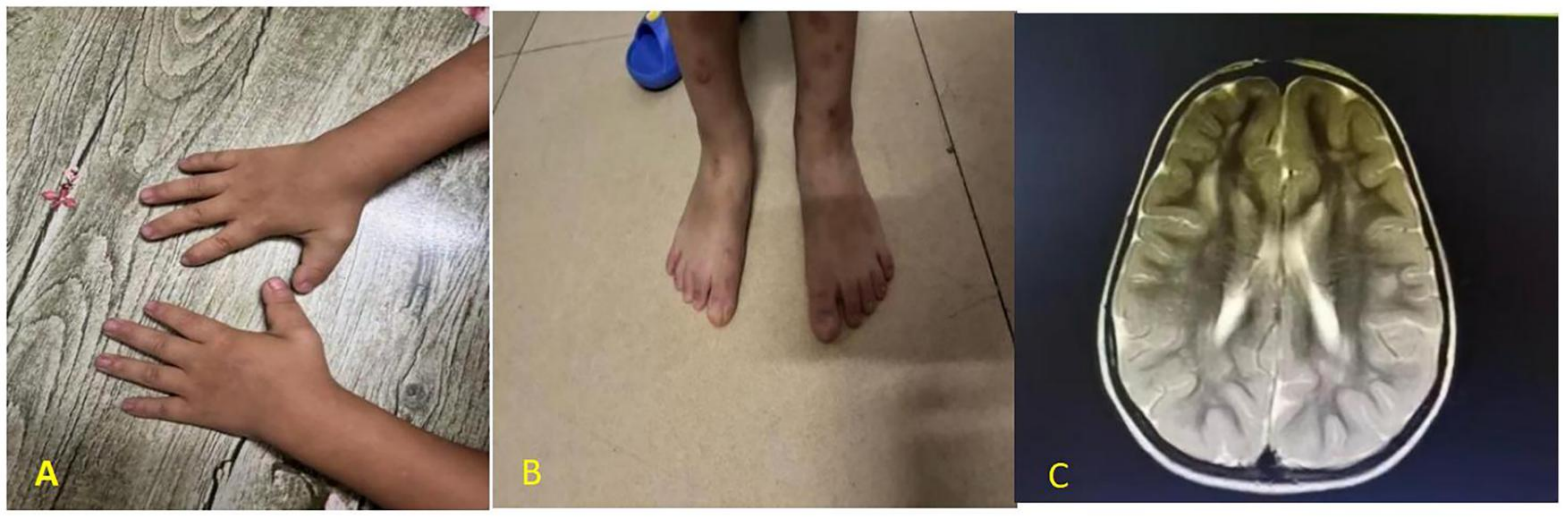
**(A)** characteristics of our pediatric patient's hands and feet (at 4 years of age); **(B)** note his small hands and feet, accompanied by brachydactyly. **(C)** Cranial MRI findings: Bilateral periventricular white matter lesions with dysmyelination.

A male newborn was admitted to the Department of Neonatology 29 min after birth due to “Born at 37 weeks of gestation with a birth weight of 2.0 kg.” His birth weight was 2.0 kg (< − 2 standard deviations [SDs]), his head circumference was 30 cm (< − 2 SDs), and his length was 45 cm (< − 1 SD). His dysmorphic facial features included a high frontal hairline, prominent forehead, hypertelorism, and a depressed nasal bridge, with normally positioned ears (Figure 1). The results of cardiorespiratory and abdominal examinations were unremarkable. Following admission, the neonate exhibited feeding difficulties and reduced spontaneous activity, which improved with non-nutritive sucking training (NNS).

### 2.2 Parental history

At the time of his birth, the mother was 24 years old and the father was 27 years old. The mother had a gravida 2 para 2 status; her first pregnancy (2017) culminated in an uncomplicated full-term vaginal delivery of a healthy male infant with a birth weight of 3.15 kg. Her past obstetric history was otherwise unremarkable. The current pregnancy was accurately dated by first-trimester ultrasound. However, prenatal surveillance revealed consistent growth delay starting at 30 + weeks of gestation, with fetal biometry measuring approximately 27 + weeks at the time. This discrepancy persisted, and a follow-up ultrasound at 32 + 6 weeks confirmed ongoing growth restriction (biometry equivalent to 29 + weeks) in the setting of oligohydramnios (Amniotic Fluid Index of 71 mm). After thorough counseling, the parents declined invasive prenatal diagnostic testing via amniocentesis.

### 2.3 Auxiliary examinations

**Figure 2:**
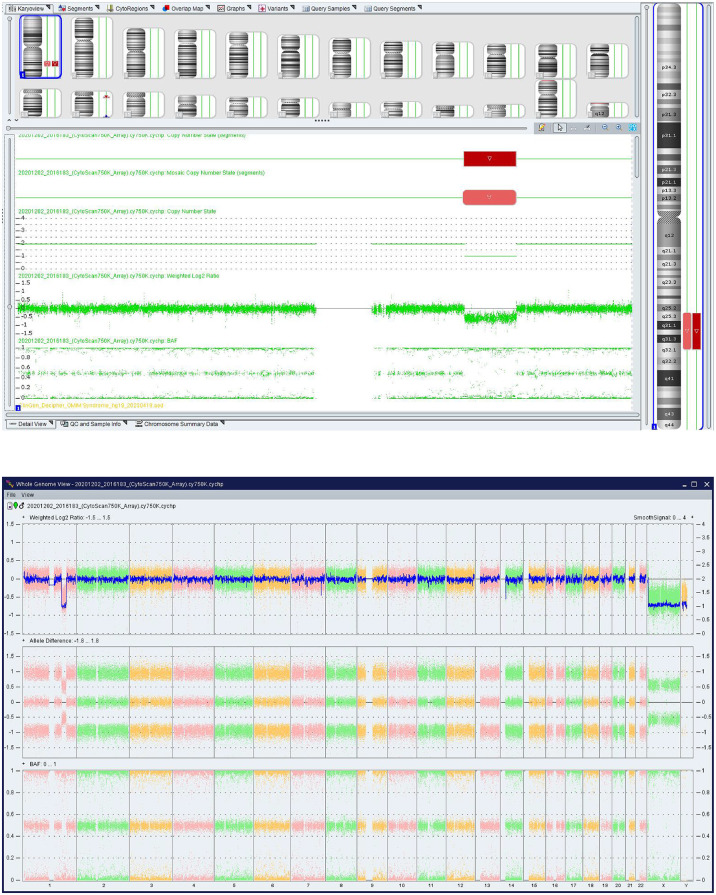
Following CMA, a pathogenic CNV was detected—specifically, a deletion at 1q25.3–q32.1. The size of the deleted segment is approximately 27.0 Mb.

Cardiac color Doppler ultrasound showed a left-to-right shunt at the atrial level, consistent with a patent foramen ovale. Cranial cavity color Doppler ultrasound revealed no abnormalities. His thyroid function triad was normal. His plasma ammonia level (via enzymatic method) was 154.50 μmol/L; meanwhile, his lactate level was 5.90 mmol/L, which returned to normal at re-examination after 72 h. Blood and urine metabolite tests showed no abnormalities. Peripheral blood karyotype analysis was performed, which indicated 46,XY,del(1)(q23.3q25.3). Further chromosomal microarray analysis (CMA) was conducted, revealing a pathogenic copy number variation (CNV)—specifically, a deletion at 1q25.3–q32.1(Chr1:180300001-207100000,hg38) with a delection size of approximately 27.0 Mb, involving 94 protein-coding genes, 88 of which were Online Mendelian Inheritance in Man (OMIM) genes (Figure 2). This test used the Affymetrix Cytoscan 750 chip (Thermo Fisher Scientific, Waltham, MA, USA) for CNV analysis, with a resolution of ≥ 100 kb for genomic CNVs and ≥ 5 Kb for regions of homozygosity. Based on the above examinations, the diagnosis was confirmed as a 1q25.3–q32.1 deletion. The parents were advised to undergo CMA, but the family refused, considering no history of hereditary diseases in the family.

### 2.4 Follow-up and clinical course

The patient received periodic rehabilitation therapy. During follow-up rehabilitation, he was diagnosed with global developmental delays, motor retardation, strabismus, and small hands and feet with brachydactyly. Although improvements in psychomotor and cognitive understanding were observed following targeted therapy, his expressive language skills remained significantly impaired. Further, the patient exhibited behavioral abnormalities, including stubbornness and a deficit in non-verbal communication skills, which precluded the establishment and maintenance of peer relationships. A cranial magnetic resonance imaging (MRI) scan at 2 months of age revealed mild corpus callosum hypoplasia. Electroencephalography findings included borderline pattern with occasional sharp waves in bilateral frontal regions during sleep. Interventions included “audiovisual stimulation training.”

At 18 months of age, his growth, motor, and language development remained delayed, with him demonstrating a height of 73 cm (< − 3 SDs), weight of 8.5 kg (< − 2 SDs), an inability to walk independently (but could with support), and an inability to say “Dad” or “Mom.” At 2 years, independent walking was achieved. Cranial MRI indicated bilateral periventricular white matter lesions with possible myelin dysplasia. Electroencephalography showed borderline patterns with slightly slowed basic rhythms during wakefulness.

At 3 years and 11 months of age, the patient's head circumference was still lagging in development. S–S language development delay assessment revealed a Group II communication attitude (poor communication attitude) and language development level of understanding stage 2-1 (functional object manipulation) with development stage 2-1. Electroencephalography revealed diagnostic features of the childhood condition on electroencephalography, such as 1) slower background activity compared to peers and 2) occasional sharp waves in the left central region during sleep. Speech therapy was initiated.

At 4 years and 11 months of age, the patient's height was 97 cm (< − 3 SDs), and his weight was 15 kg (< − 3 SDs). He was able to walk and run but showed significant language delays and poor imitation skills. He was enrolled in a special education school at the time of last follow-up.

## 3 Literature review

### 3.1 Literature search and selection criteria

A comprehensive literature search was conducted in the PubMed, Embase, China National Knowledge Infrastructure, and Wanfang electronic literature databases to identify relevant publications from January 1976 to January 2025. The search terms of interest included: “1q deletion,” “1q25 deletion,” “1q25–q32 interstitial deletion,” and “chromosome 1q deletion in neonate.” The inclusion criteria were as follows: 1) patients with a confirmed clinical diagnosis of 1q25–q32 deletion; 2) availability of complete clinical data; and 3) confirmation of the deletion's location and size by karyotyping, CMA, or whole-genome sequencing. Separately, 1) cases with concomitant chromosomal abnormalities (e.g., translocations, other deletions/duplications, or aneuploidies such as trisomy 21), 2) cases with incomplete clinical documentation, and 3) studies without full-text versions available were excluded.

**Figure 3:**
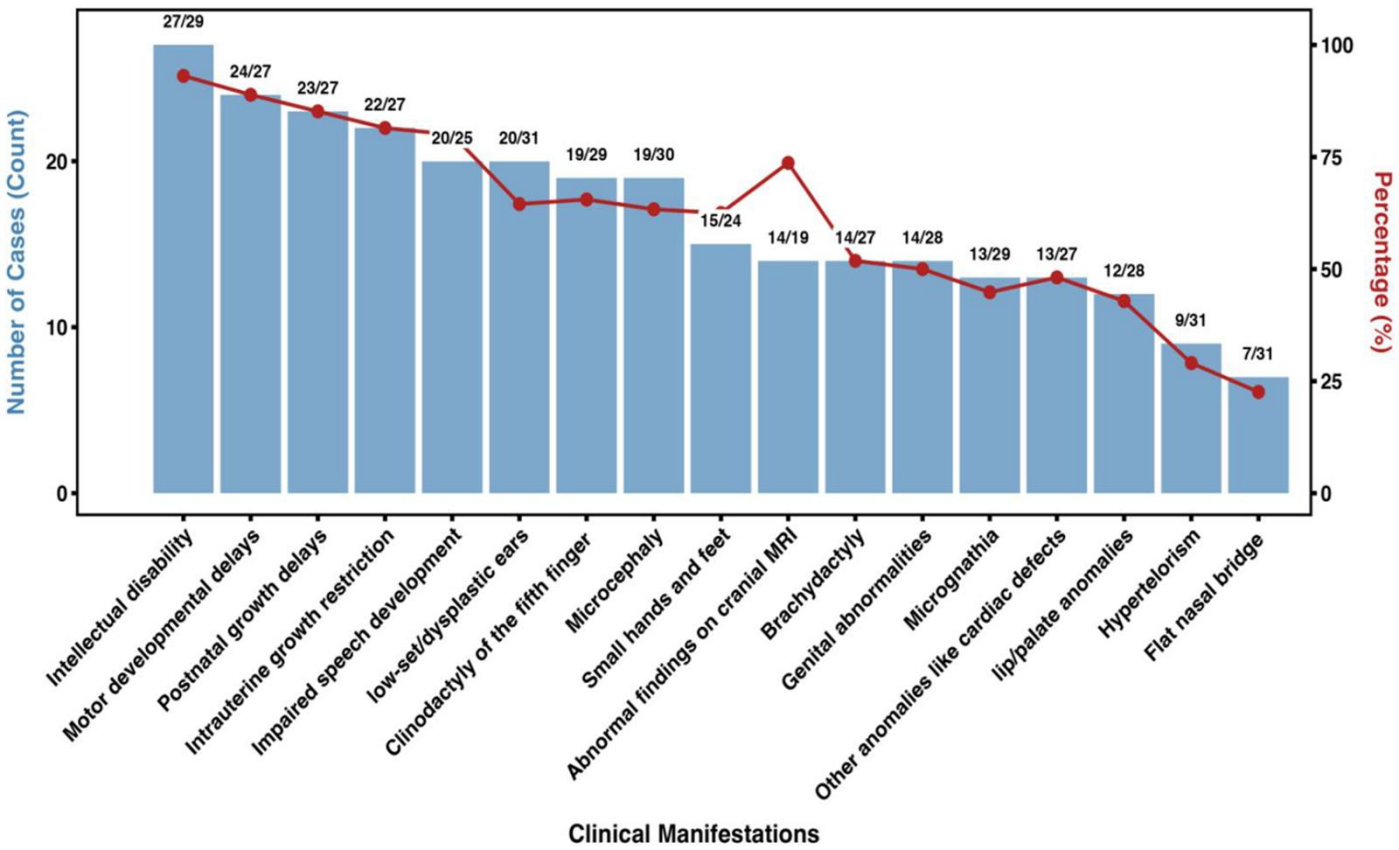
Frequency of clinical manifestations in patients with 1q25.3–q32.1 deletion syndrome. Blue bars represent the absolute number of cases with each clinical feature (left *y*-axis). Red line with markers shows the corresponding percentage of affected patients for each manifestation (right *y*-axis, %). The fraction above each data point indicates the number of affected cases over the total number of patients evaluated for that specific feature. Clinical features are ordered from most to least prevalent.

A total of 31 reported cases were included in the final analysis, including five cases reported from China. A summary of the clinical features is presented in Table 1. The cohort included 16 males (51.6%) and 13 females (41.9%), with sex being undetermined in two cases (6.5%), indicating a relatively balanced sex distribution. The genomic analysis identified 1q25–q32 as the most common deletion interval. In the cohort, deletion sizes averaged 16.19 Mb and ranged from 1.5 to 28.025 Mb. Most genetic sources of 1q25–q32 deletion were *de novo* (16/22). The main diagnostic methods included karyotype analysis, CMA, and whole-genome sequencing. The spectrum of common clinical manifestations included the following: 1) growth abnormalities, such as intrauterine growth restriction (22/27), postnatal growth delays (23/27), and microcephaly (19/30); 2) facial dysmorphisms, such as low-set/dysplastic ears (20/31), micrognathia (13/29), hypertelorism (9/31), a flat nasal bridge (7/31), and lip/palate anomalies (12/28); 3) neurological involvement, such as intellectual disability (27/29), motor developmental delays (24/27), impaired speech development (20/25), and abnormal findings on cranial MRI (14/19); and 4) other anomalies like cardiac defects (13/27), clinodactyly of the fifth finger (19/29), genital abnormalities (14/28), brachydactyly (14/27), and small hands and feet (15/24) (Figure 3). Other reported features included hypothyroidism, growth hormone deficiency, renal malformations, a prominent forehead, a high frontal hairline, sparse hair, strabismus, a short/bulbous nose, and brain developmental malformations.

**Table 1:** Comparison of clinical phenotypes in children with 1q25.3–q32.1 deletion.

Number	Case report	Sex	Deletion location	Size of deletion (Mb)	Intrauterine growth restriction	Growth and developmental delays	Motor disorder	Intellectual disability	Language developmental disorder	Cleft lip/palate	Hypertelorism	Low and Flat nose bridge	Abnormal ear position or ear malformation	Microcephaly	Micrognathia	Small hands/feet	Brachydactyly	Clinodactyly of the fifth finger	Cardiac defect	Genital abnormality	Abnormal cranial/brain MRI results
1	This case	Male	1q25.3–q32.1	27	+	+	+	+	+	−	+	+	−	+	+	+	+	−	−	−	+
2	Yu et al. (6)	Female	1q25.1–q31.1	14.86	+	+	+	+	+	−	+	+	−	+	−	−	−	−	+	−	+
3	Song et al. (5)	ND	1q23.3–q31.2	28.025	+	ND	ND	ND	ND	+	−	−	−	−	−	−	−	−	+	−	+
4	Yirou et al. (4)	Male	1q24.3–q25.3	14.27	+	+	+	+	+	−	+	+	+	−	+	+	+	−	−	+	＋
5	Chatron et al. (7)	Female	1q25.1–q25.3	7.12	+	+	+	+	+	−	+	−	+	+	−	+	−	+	−	−	ND
6	Chatron et al. (7)	Female	1q25.1–q25.3		+	+	−	+	−	−	−	−	+	+	−	+	+	−	ND	−	ND
7	Chatron et al. (7)	Female	1q25.2–q25.3	6.16	ND	+	−	+	−	−	−	−	−	+	−	+	−	+	+	−	ND
8	Chatron et al. (7)	ND	1q24.3–q31.2	20.95	ND	+	−	+	−	+	−	−	+	−	+	+	+	+	−	+	ND
9	Libotte et al. (8)	Male	1q25.3–q32.1	18	+	ND	ND	ND	ND	−	−	−	+	−	−	−	−	−	−	−	ND
10	Hu et al. (9)	Female	1q25.2–q31.3	20.5	−	−	+	+	+	-	+	+	−	−	−	−	−	＋	−	−	−
11	Milani et al. (10, 11)	Male	1q31.1–q32.1	15.6	−	−	+	+	+	+	−	−	+	−	−	−	−	−	−	−	−
12	Filges et al. (11, 12)	Female	1q25.2–q25.3	1.5	+	+	−	−	−	−	−	−	−	−	−	−	−	−	+	−	+
13	Yan et al. (12)	Female	1q25.1–q31.3	20.561	−	−	+	+	+	−	+	−	−	−	−	−	−	＋	+	−	+
14	Burkardt et al. (13)	Male	1q24.3–q25.2	12.48	+	+	+	+	+	−	+	−	+	+	−	+	+	+	−	+	+
15	Burkardt et al. (13)	Male	1q24.1–q31.1	26.7	+	+	+	+	+	+	−	−	+	+	−	−	+	−	+	+	−
16	Burkardt et al. (13)	Male	1q24.3–q25.3	9.81	+	+	+	+	+	+	−	−	−	+	+	+	−	+	−	−	+
17	Burkardt et al. (13)	Female	1q24.3–q25.3	12.9	+	+	+	+	+	−	−	−	+	+	−	+	+	+	−	+	+
18	Burkardt et al. (13)	Male	1q24.3–q31.3	22.3	+	+	+	+	+	+	−	−	+	+	+	+	−	+	+	+	−
19	Nishimura et al. (14)	Male	1q24.3–q31.2	19.5	+	+	+	+	+	+	−	−	−	＋	－	+	+	+	－	+	+
20	Thienpont et al. (15)	Male	1q25.1–q31.3	20.3	+	+	+	+	+	+	+	−	−	−	−	+	−	+	−	−	−
21	Descartes et al. (16)	Female	1q23.3–q25.2	14.38	−	+	+	+	+	+	−	−	+	+	+	+	+	+	+	+	+
22	Schwemmle et al. (17)	Male	1q23–q31	ND	+	+	+	+	+	＋	−	+	+	+	+	ND	ND	+	+	−	ND
23	Hoglund et al. (18)	Male	1q25.3–q31.3	13.1	−	−	+	−	+	−	−	−	+	−	+	−	+	−	−	+	ND
24	Pallotta et al. (19)	Male	1q23–q31.2	ND	+	+	+	+	−	+	−	−	+	−	+	+	+	+	+	−	+
25	Scarbrough et al. (20)	Male	1q25–q32		+	ND	ND	+	ND	−	−	+	+	+	+	ND	ND	+	ND	+	+
26	Steinbach et al. (21)	Male	1q25.2–q31.2		+	+	+	+	+	−	−	−	+	+	+	ND	ND	+	+	+	+
27	Taysi et al. (3)	Female	1q23–q25		+	+	+	+	+	+	−	−	+	+	−	+	−	+	+	+	ND
28	Pan et al. (22)	Female	1q25–q31		+	ND	ND	+	ND	+	−	−	+	+	ND	ND	+	+	+	ND	+
29	Garver et al. (23)	Female	1q25–q32		ND	+	+	+	ND	ND	−	−	+	+	+	ND	+	+	−	ND	ND
30	Garver et al. (23)	Female	1q25–q32		ND	+	+	+	ND	ND	−	−	+	+	+	ND	+	+	+	ND	ND
31	Koivisto et al. (24)	Male	1q25–q32		+	+	+	+	+	ND	+	+	−	ND	ND	ND	ND	ND	ND	+	ND

+, yes; −, no; CMA, chromosomal microarray analysis; MRI, magnetic resonance imaging; ND, undescribed; SNP, single, nucleotide polymorphism array; WGS, whole, genome sequencing.

## 4 Discussion

A review of the literature indicates that the 1q25.3–q32.1 deletion is a mid-segment deletion of chromosome 1. However, in 1980, Schinzel and Schmid (25) first described a recognizable proximal 1q-deletion syndrome characterized by severe pre- and postnatal growth failure; profound mental deficiencies; a pattern of dysmorphic stigmata involving mainly the face and distal limbs; bilateral cleft lip and cleft palate; and peculiar radiologic findings of the hands and feet, especially short metacarpals, metatarsals, and phalanges as well as cone-shaped epiphyzes and bifid terminal phalanges of the thumbs and halluces. We are not aware of any other skeletal dysplasia with a similar pattern of x-ray findings (25). The primary clinical manifestations summarized from the identified cases include intellectual disability, intrauterine and postnatal growth retardation, motor developmental delays, language developmental delays, microcephaly, low-set or malformed ears, micrognathia, hypertelorism, a depressed nasal bridge, a broad/bulbous nasal tip, a prominent forehead, a high frontal hairline, sparse hair, strabismus, a short nose with a bulbous tip, cleft lip/palate, cardiac defects, clinodactyly of the fifth finger, genital abnormalities, brachydactyly, small hands and feet, feeding difficulties, and renal malformations. Additionally, hypothyroidism, growth hormone deficiency, and abnormal cranial MRI findings may also be documented. The clinical manifestations of this disorder are instead highly dependent on the specific genes involved in the deletion, and no correlation was observed between the size of the deletion and the severity of the clinical phenotype (7). Burkardt et al. (13) reported a case with a 12.9-Mb deletion characterized by severe growth retardation and profound intellectual disability. In contrast, the case 10, carrying a larger 20.5Mb deletion, exhibited a significantly milder clinical phenotype. Our patient presented solely with intellectual disability, clinodactyly of the fifth finger, and mild facial dysmorphism, without notable growth retardation (9), suggesting that whether critical genes are involved or not has a greater impact on phenotypic severity than the size of the deletion. Most gene deletions are *de novo* in origin, with a minority being of parental origin. Adult females have fertility potential and can transmit these gene deletions to their offspring (13). Diagnostic methods primarily include chromosome karyotype analysis, CMA, array-based comparative genomic hybridization, and whole-genome sequencing.

**Figure 4:**
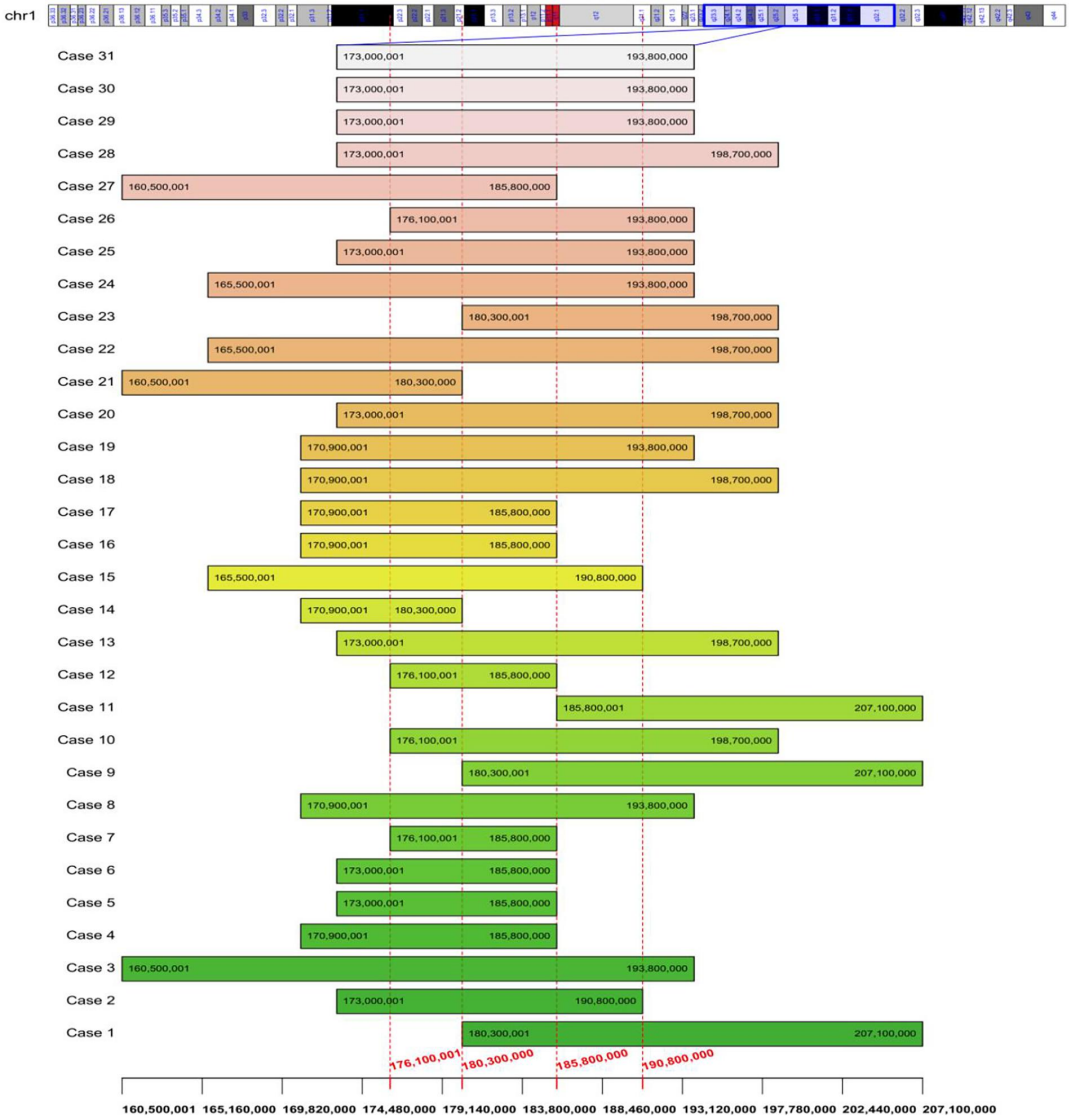
The figure presents a cytogenetic ideogram of chromosome 1, where the centromere is highlighted in red and cytobands are labeled in blue. The blue blocks within the ideogram represent observed CNVs in the 31 cases from the literature. Individual CNV events are depicted as colored boxes, with their specific genomic coordinates annotated on either side. Red dashed lines delineate the minimal overlap regions among cases. Meanwhile, the segments defined between consecutive dashed lines represent shared genomic intervals common to a specific subset of cases. All genomic coordinates are based on the GRCh38/hg38 reference genome. The slight differences in deletion size and coordinates between Figure 4 and Table 1 are due to the unification of all genomic intervals to GRCh38/hg38 in this figure, while the original data in Table 1 were derived from published studies using different earlier genome assemblies.

Chromosomal genetic data for all cases were integrated, and their genomic coordinates were precisely mapped based on the Hg38 genome assembly and visualized in Figure 4. Our analyses revealed that the deletion segments identified in the 31 cases spanned the interval from 1q23.3 to 1q32.1. With the exception of case 11 (involving a deletion of 1q31.1-q32.1), the deletion regions of the remaining cases do not fully overlap; however, by focusing on the most highly overlapping segments, the minimal common region was delineated to be 1q25.2-q25.3. This segment is recurrently involved in the vast majority of published cases, suggesting that it likely harbors key genes underlying core clinical phenotypes, including growth retardation, developmental delays, and intellectual disability. The distribution of breakpoints revealed that the most frequent start points clustered at 1q24.3 and 1q25.1, while the most frequent end points clustered at 1q25.3 and 1q32.1. This clustering of breakpoints within specific sub-bands indicates that these genomic regions may be structurally unstable and prone to chromosomal breakage.

Notably, the patient in case 12 (11), with a deletion as small as 1.5 Mb (1q25.2–q25.3), still presented with typical growth and developmental delays, strongly indicating that this region contains critical causative genes. Integrating relevant literature findings with high-resolution genomic annotation of this microdeletion interval, we confirmed that this locus contains at least two genes—*LHX4* and *FAM20B* (26)—that are associated with development, with *LHX4* likely being the core pathogenic gene.

The present study found that the 1q24–q32 deletion encompasses numerous genes, including *CENPL*, *LHX4*, *PBX1*, *DNM3*, *ASTN1*, and *FAM20B* (5, 7, 9, 13). Of these, *PBX1*, *CENPL,* and *LHX4* remain the primary candidate genes for growth retardation (5). *CENPL* (1q25.1; chr1:173799550–173824883, hg38) encodes centromere protein L (a subunit of the CENPA–CAD complex) (27), which targets the CENP-A chromatin domain to the centromere and is crucial for normal kinetochore function and mitotic progression (28). Therefore, the deletion or mutation of genes encoding centrosomal proteins, such as *CENPL*, may underlie the primordial growth deficiency and severe microcephaly observed in 1q24q25-deletion syndrome (13). The *LHX4* (chr1:180230264–180278984, hg38) gene is located in the 1q25 region; belongs to the LIM homeobox gene family; and regulates pituitary cell differentiation and development, particularly affecting the secretion of growth hormone and thyroid-stimulating hormone (24). The LHX4 protein coordinates the development of the pituitary region and the underlying sphenoid bone during cranial morphogenesis (29). Heterozygous mutations in *LHX4* lead to a phenotype of short stature, anterior pituitary hormone deficiency, and abnormalities of the brain pituitary and cerebellum (30). Koivisto et al. (24) were the first to observe primary hypothyroidism and growth hormone deficiency in a child with a 1q25–q32 deletion. It is noteworthy that the neonate in this case had normal thyroid function, which is hypothesized to be because the core coding region is located at 1q25.2 and their deletion did not involve the causative gene, hence the normal thyroid function. Meanwhile, Burkardt et al. (13) proposed *DNM3* (1q24.3; chr1:171841498–172412717, hg38) as a candidate gene associated with intellectual disability. *DNM3* possesses mechanochemical properties, participates in actin–membrane processes, and interacts with the Homer–Shank scaffold complex postsynaptically in hippocampal neurons (31). In 1q24q25-microdeletion syndrome, haploinsufficiency of the *DNM3* gene may lead to delayed myelination and other neurodevelopmental disorders; haploinsufficiency of the miRNA within its intron may contribute to skeletal and dysmorphic features (13). *ASTN1*1q25.2(chr1:176861067-177164712，hg38) (astrocytic cell adhesion molecule 1) plays a critical role in glial-guided neuronal migration within the cerebral cortex (32). Mutations in *ASTN1* can lead to a spectrum of cortical malformations, including lissencephaly, pachygyria, abnormal cortical thickening, lateral ventricular enlargement, and hypoplasia of the corpus callosum or cerebellum (32). Heterozygous deletion of the *ASTN1* gene may serve as a key etiological factor for intellectual disability in patients with 1q25–32 deletions (9).

Chromosome karyotyping, a cost-effective technique, detects chromosomal numerical and structural abnormalities by analyzing morphological features, including chromosome length, centromere position, and arm ratio, thereby clarifying the etiology of genetic disorders. However, this method has inherent limitations: its resolution is only above 5 Mb, rendering it incapable of detecting microstructural anomalies. In this study, no chromosomal deletions were identified in cases 11 and 12, which may be attributed to technical experience and chromosome size. In particular, the 1.5-Mb deletion in case 12 was more likely to be missed due to its small size. CMA, in contrast, is a high-resolution detection technique with a resolution ranging from 10 to 100 kb, enabling genome-wide screening for aneuploidy, microduplications, and microdeletion syndromes (5). Based on this, the American College of Obstetricians and Gynecologists has explicitly recommended CMA as a first-line diagnostic tool for fetal structural abnormalities (33).

Currently, there are no specific treatments for 1q25-q32 deletions, and clinical management primarily focuses on symptomatic treatment and long-term follow-up. Specific recommendations are as follows:

- *Growth and endocrine management.* Regularly monitor growth parameters (weight, length, head circumference) and endocrine function (e.g, growth hormone, thyroid function). For cases definitively diagnosed with growth hormone deficiency, growth hormone replacement therapy may be considered. However, based on our literature review, some cases show a poor response to growth hormone therapy (4, 13), suggesting that their growth retardation may result from multiple factors, and responses to growth hormone treatment show individual variation.
- *Neurorehabilitation intervention.* Implement early rehabilitation therapy for children with motor and language developmental delays to improve quality of life. However, long-term neurodevelopmental outcomes vary significantly, typically requiring ongoing rehabilitation support and special education.
- *Genetic counseling.* Comprehensive genetic counseling should be provided to the family. If both parents consent and undergo CMA, such can determine whether the deletion is a *de novo* mutation or inherited, thereby suggesting the recurrence risk and providing a basis for prenatal diagnosis in subsequent pregnancies.

The prognosis of 1q25-q32 deletion is associated with the involvement of key genes and the severity of multiorgan involvement: cases presenting only with mild growth retardation and no severe organ malformations have a relatively favorable prognosis, while those with severe cortical maldevelopment, refractory seizures, or multiple severe organ malformations have a poor prognosis, with some cases resulting in infant mortality (20). Clinical management should emphasize multidisciplinary collaboration (including neonatology, genetics, endocrinology, and rehabilitation medicine), encompassing regular monitoring of growth and endocrine function, early rehabilitation intervention, and genetic counseling. Accumulation of more cases and long-term follow-up studies are necessary to further elucidate the genotype–phenotype correlation and improve patient outcomes.

## Data Availability

The original contributions presented in the study are included in the article/Supplementary Material, further inquiries can be directed to the corresponding author.
