# Supplementary material for: 1q25.3–q32.1 deletion causing multisystem developmental delay: a case report and literature review
Source: Front Pediatr. 2026 Jun 10;14:1774631. doi: 10.3389/fped.2026.1774631 (PMC13290558; doi:10.3389/fped.2026.1774631)
Supplement: Supplementary file 1 [file Supplementaryfile1.docx]

Supplementary information(SI)

1q25.3–q32.1 Deletion Causing Multisystem Developmental Delay: A Case Report and Literature Review

**A:FIGURE 5.** Karyotype analysis revealed 46,XY,del(1)(q23.3q25.3).


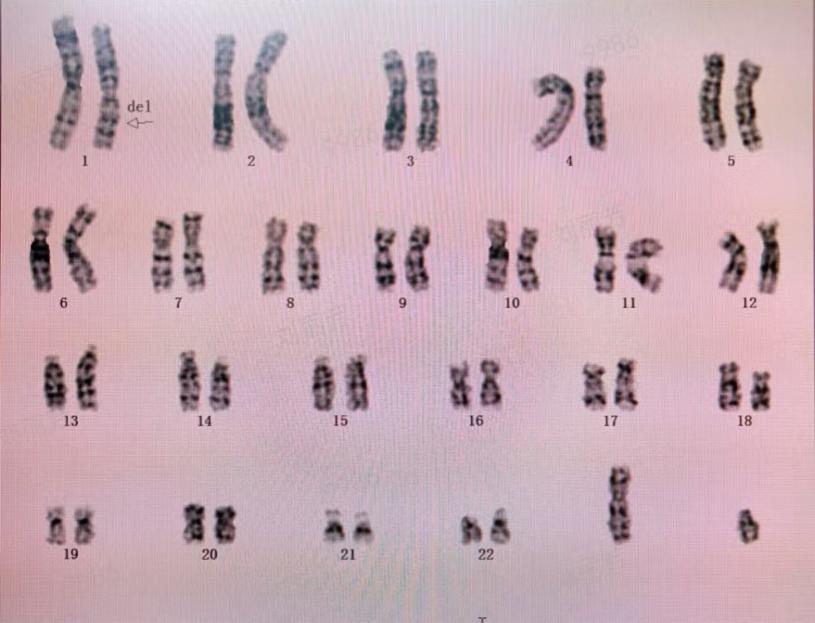


**B:TABLE2:The start and end breakpoints of the deletion segment in a single case were identified in accordance with the hg38 genome assembly.**

| case | Cytogenetic band location | hg38 genome chr1 start coordinate | hg38 genome chr1 end coordinate |
| --- | --- | --- | --- |
| 1 | 1q25.3–q32.1 | 180,300,001 | 207,100,000 |
| 2 | 1q25.1–q31.1 | 173,000,001 | 190,800,000 |
| 3 | 1q23.3–q31.2 | 160,500,001 | 193,800,000 |
| 4 | 1q24.3–q25.3 | 170,900,001 | 185,800,000 |
| 5 | 1q25.1–q25.3 | 173,000,001 | 185,800,000 |
| 6 | 1q25.1–q25.3 | 173,000,001 | 185,800,000 |
| 7 | 1q25.2–q25.3 | 176,100,001 | 185,800,000 |
| 8 | 1q24.3–q31.2 | 170,900,001 | 193,800,000 |
| 9 | 1q25.3–q32.1 | 180,300,001 | 207,100,000 |
| 10 | 1q25.2–q31.3 | 176,100,001 | 198,700,000 |
| 11 | 1q31.1–q32.1 | 185,800,001 | 207,100,000 |
| 12 | 1q25.2–q25.3 | 176,100,001 | 185,800,000 |
| 13 | 1q25.1–q31.3 | 173,000,001 | 198,700,000 |
| 14 | 1q24.3–q25.2 | 170,900,001 | 180,300,000 |
| 15 | 1q24.1–q31.1 | 165,500,001 | 190,800,000 |
| 16 | 1q24.3–q25.3 | 170,900,001 | 185,800,000 |
| 17 | 1q24.3–q25.3 | 170,900,001 | 185,800,000 |
| 18 | 1q24.3–q31.3 | 170,900,001 | 198,700,000 |
| 19 | 1q24.3–q31.2 | 170,900,001 | 193,800,000 |
| 20 | 1q25.1–q31.3 | 173,000,001 | 198,700,000 |
| 21 | 1q23.3–q25.2 | 160,500,001 | 180,300,000 |
| 22 | 1q23–q31 | 165,500,001 | 198,700,000 |
| 23 | 1q25.3–q31.3 | 180,300,001 | 198,700,000 |
| 24 | 1q23–q31.2 | 165,500,001 | 193,800,000 |
| 25 | 1q25–q32 | 173,000,001 | 193,800,000 |
| 26 | 1q25.2–q31.2 | 176,100,001 | 193,800,000 |
| 27 | 1q23–q25 | 160,500,001 | 185,800,000 |
| 28 | 1q25–q31 | 173,000,001 | 198,700,000 |
| 29 | 1q25–q32 | 173,000,001 | 193,800,000 |
| 30 | 1q25–q32 | 173,000,001 | 193,800,000 |
| 31 | 1q25–q32 | 173,000,001 | 193,800,000 |
